# Editorial: Pharmacotherapy of Anxiety Disorders: Promises and Pitfalls

**DOI:** 10.3389/fpsyt.2021.662963

**Published:** 2021-03-18

**Authors:** Amir Garakani, Rafael C. Freire, James W. Murrough

**Affiliations:** ^1^Department of Psychiatry, Icahn School of Medicine at Mount Sinai, New York, NY, United States; ^2^Silver Hill Hospital, New Canaan, CT, United States; ^3^Department of Psychiatry, Yale University School of Medicine, New Haven, CT, United States; ^4^Department of Psychiatry and Centre for Neuroscience Studies, Queen's University, Kingston, ON, Canada; ^5^Department of Neuroscience, Icahn School of Medicine at Mount Sinai, New York, NY, United States

**Keywords:** anxiolytic, novel medications, panic, social anxiety, specific phobia, generalized anxiety, PTSD, psychopharmacology

Despite being among the most common psychiatric disorders worldwide, and a leading cause of disability including work and school absences, anxiety disorders have been relatively under-represented in recent research of novel pharmacologic agents, compared to major depressive disorder (MDD) and schizophrenia. Panic disorder (PD), generalized anxiety disorder (GAD), and social anxiety disorder (SAD) are commonly treated with either medications and/or psychotherapy, while specific phobias (SP) are usually treated with behavioral therapy alone. While there is support for certain forms of psychotherapy to treat anxiety disorders, there remains concern about lower efficacy of psychotherapies compared to medications (1), and incomplete treatment response, and evidence that patients with certain anxiety disorders, especially GAD and SAD, have high rates of recurrence (2, 3). Most research of medications of anxiety disorders have been focused on the gamma aminobutyric acid (GABA), serotonin and norepinephrine systems. The first-line medications approved by the United States Food and Drug Administration (FDA) for treatment of PD, GAD, and SAD are selective serotonin reuptake inhibitors (SSRIs) and serotonin norepinephrine reuptake inhibitors (SNRIs) while benzodiazepines, which are GABA-A receptor agonists, are also approved for treatment either as monotherapy or adjunctive treatment for anxiety (4–7). Despite the promise of newer serotonergic agents, or antipsychotics, or GABAergic drugs (like pregabalin and gabapentin), there has not been an FDA-approved medication for anxiety disorders since duloxetine, an SNRI, was approved for GAD in 2007 (8).

The purpose of this Research Topic was to collect original papers and review articles exploring promising novel medications on the pipeline for anxiety disorders, primarily GAD, PD, SAD and SP, after first reviewing the current state of psychopharmacological treatments available. The topic aimed to explore more unique pathways for targeting treatment response in anxiety disorders, including the glutamate system, neurosteroids, the hypothalamic-pituitary-adrenal (HPA axis), neuropeptides, cannabinoids, and phytochemicals.

Lijffijt et al. reported a protocol for a randomized, placebo-controlled proof-of-mechanism trial of a n-methyl-d-aspartate (NMDA) receptor antagonist lanicemine for 24 adults with symptoms of post-traumatic stress disorder (PTSD) (as measured by the Clinician Administered PTSD Scale (CAPS)]. The study was included in this Research Topic despite being a trial on participants with PTSD symptoms because of its potential application to anxiety disorders. In the protocol, participants are to receive 5 days of intravenous (IV) injections of lanicemine or placebo and be monitored with anxiety-potentiated startle and CAPS scores. Lijffijt et al. are building upon previous research of another NMDA antagonist, ketamine, which has also been tested using IV infusions in patients with treatment-resistant depression (9–13), comorbid PTSD and MDD (14), and randomized controlled trials showing potential efficacy in PTSD as well (15, 16). There will be great interest in the results of this clinical study when it is concluded.

Given the heightened interest in NMDA receptor antagonists for depression and PTSD, Nasir et al. provided a review of glutamate, the principal excitatory neurotransmitter of the central nervous system (CNS), and its interactions with GABA, the primary CNS inhibitor, in anxiety disorders. Building from an overview of circuitry and receptor pathways, and a review of preclinical research describing the role glutamate plays in anxiety, Nasir et al. then describe the current state of glutamatergic and GABAergic drug research in anxiety disorders. The GABA modulators discussed in the paper, most of which are clinically used as anticonvulsants (levetiracetam, topiramate, tiagabine, and valproic acid), have limited support due the absence of larger, randomized, double-blind, placebo-controlled trials, with the exception of pregalabin, which was approved for GAD in Europe in 2006 (8). Glutamate modulators like ketamine, memantine, d-cycloserine, n-acetylcysteine, and riluzole, have shown promise in open-label or small, controlled trials but there are few if any larger-scale studies.

Understanding stress response and hormone regulation may provide further clarity about newer pharmacological treatments, and Tafet and Nemeroff explored how the HPA axis plays a role in anxiety and stress. The review paper discusses the neurobiology of the HPA axis, serotonergic and norepinephrine systems and how traditional treatments for anxiety disorders, like tricyclic antidepressants (TCAs), SSRIs, and benzodiazepines may normalize hyperactivity of the HPA axis. Tafet and Nemeroff cite evidence that these antidepressants may modulate glucocorticoid receptors in the brain and benzodiazepines may have an inhibitory effect on corticotrophin releasing factor (CRF). Taken together, the findings support an expansion of research beyond serotonin and norepinephrine and may spur exploration of novel agents targeting the HPA axis directly.

Finally, the Topic Editors, Garakani et al., along with other authors, presented an overview of the current state of treatment of anxiety disorders, which is discussed above, followed by a review of novel pharmacologic treatments for PD, GAD, SAD, and SP. Several serotonergic agents that were originally investigated for depression have been studied and showed potential, but these trials did not lead to FDA approval and there are limited ongoing investigations. There are also trials of psilocybin and lysergic acid diethylamide (LSD) for anxiety but primarily in those with life-threatening diseases (like cancer) (17, 18). Ketamine has been studied in a randomized controlled trial in SAD but there are no ongoing trials in anxiety disorders (19). Studies of d-cyloserine in anxiety have mostly been negative. There are few neuropeptides under study for anxiety as well and trials of CRF antagonists have not shown efficacy in anxiety disorders. Research on cannabinoids for anxiety have also been disappointing and shown possible worsening of symptoms, particularly with Delta-9-tetrahydrocannabinol (THC). The primary areas of promise are the neurosteroid PH94B, an intranasally administered aerosol, which underwent two randomized controlled trials for acute SAD (20, 21), and the growing research on phytochemicals including kava.

Taken as a whole, the Research Topic did in fact show both promises and disappointments in the research of novel pharmacotherapeutics for anxiety disorders. As expected, the pathways and novel compounds hypothesized to treat anxiety were hampered by lack of efficacy (perhaps due to poor study design), small sample sizes or other mitigating factors. With any hope, this Topic should not discourage ongoing exploration beyond serotonin, norepinephrine and GABA, and instead bolster the efforts to expand on our understanding of the complex neurobiological pathways of anxiety and how to translate this work into novel agents that perhaps can complement the ongoing research into psychotherapeutic treatments of anxiety.

## Author Contributions

AG wrote the original draft of the manuscript. AG, RF, and JM contributed to critical manuscript revisions. All authors reviewed and approved the final draft of the manuscript and made substantial contributions to this study.

## Conflict of Interest

JM disclosures: In the past 5 years, JM has provided consultation services and/or served on advisory boards for Allergan, Boehreinger Ingelheim, Clexio Biosciences, Fortress Biotech, FSV7, Global Medical Education (GME), Impel Neuropharma, Janssen Research and Development, Medavante-Prophase, Novartis, Otsuka, and Sage Therapeutics. JM was named on a patent pending for neuropeptide Y as a treatment for mood and anxiety disorders and on a patent pending for the use of ezogabine and other KCNQ channel openers to treat depression and related conditions. The Icahn School of Medicine (employer of JM) was named on a patent and has entered into a licensing agreement and will receive payments related to the use of ketamine or esketamine for the treatment of depression. The Icahn School of Medicine is also named on a patent related to the use of ketamine for the treatment of PTSD. JM was not named on these patents and will not receive any payments. The remaining authors declare that the research was conducted in the absence of any commercial or financial relationships that could be construed as a potential conflict of interest.
